# Family Care Partners’ Daily Affect and Sleep in Dementia Care Dyads Using Ecological Momentary Assessment

**DOI:** 10.1093/geroni/igaf122.311

**Published:** 2025-12-31

**Authors:** Yeonsu Song, Raeanne Moore, Brent Mausbach, Jennifer Martin, Mary-Lynn Brecht

**Affiliations:** University of California, Los Angeles, Los Angeles, California, United States; University of California, San Diego, San Diego, California, United States; University of California, San Diego, San Diego, California, United States; VA Greater Los Angeles Healthcare System, Los Angeles, California, United States; University of California, Los Angeles, Los Angeles, California, United States

## Abstract

Poor sleep is associated with lower positive affect and higher negative affect. Many family care partners (CP) of people living with dementia (PLWD) experience poor sleep. However, the impact of poor sleep on daily affect among CP, and the reverse relationship, remains unclear. This study examined daily variations in positive and negative affect and their relationship with poor sleep using data from an ongoing clinical trial involving dementia care dyads. A total of 72 CP completed ecological momentary assessment surveys of their positive and negative affect (three times a day) and sleep of CP and PLWD for seven days. Variables included positive (feeling energetic, happy, motivated, rested, relaxed) and negative affect (feeling anxious, depressed, helpless, hopeless, nervous, tense, worried, worthless), and ‘good sleep’ (no trouble falling asleep and staying asleep) on previous nights. Descriptive and mixed-effects regression models were conducted. Positive affect was higher in the morning compared to midday and evening. Negative affect was lower in the evening than the morning, and higher at midday than in the morning. The relationship between positive affect and the previous night’s CP sleep differed significantly between the morning and the evening (β = 0.079, p = 0.049). In a lagged mixed-effect model, negative affect was significantly related to the next day’s sleep for both CP and PLWD. Higher levels of negative affect were associated with a greater likelihood of poor sleep for both dyad members (β = 1.435, p = 0.007 for CP; β = 1.398, p = 0.038 for PLWD). Understanding daily affect fluctuations is essential for improving sleep among dementia care dyads.

